# The relationship between shoulder pain and shoulder disability in women: The mediating role of sleep quality and psychological disorders

**DOI:** 10.1097/MD.0000000000031118

**Published:** 2022-10-14

**Authors:** Younghui Hwang, Jihyun Oh

**Affiliations:** a Department of Nursing Science, College of Medicine, Chungbuk National University, Cheongju, Korea; b Department of Nursing, College of Nursing and Health, Kongju National University, Kongju, South Korea.

**Keywords:** anxiety, depression, shoulder disability, shoulder pain, sleep quality, stress

## Abstract

The number of patients complaining of shoulder pain is gradually increasing, and women with shoulder pain in particular tend to present with psychological disorders and poor sleep quality. Therefore, the purpose of this study was to investigate whether psychological disorder and poor sleep quality mediated the relationship between shoulder pain and shoulder disability in women. This is a descriptive survey study of 222 women from 3 community centers in Seoul and Gyeonggi-do regions in South Korea. Data about shoulder pain, shoulder disability, sleep quality, depression, anxiety, and stress were collected using structured questionnaires between May 10 and June 7, 2021, and were analyzed by serial mediation analysis. The direct effect of shoulder pain on shoulder disability was statistically significant. Serial mediation model showed that sleep quality and psychological disorders (depression, anxiety, and stress) were sequential mediators between shoulder pain and shoulder disability among women. The relationship between shoulder pain and shoulder disability among women was partially mediated by sleep quality and psychological disorders (depression, anxiety, and stress). The results emphasize the importance of sleep quality and psychological factors in shoulder disability and suggest the use of strategies to improve sleep quality and alleviate psychological factors when developing an intervention program to mitigate shoulder disability in women with shoulder pain.

## 1. Introduction

Shoulder pain is one of the most frequent complaints involving the musculoskeletal system.^[1]^ Shoulder pain occurs in or around the shoulder and stems from the joint and surrounding soft tissues.^[2]^ The average incidence rate of shoulder pain in general practice from 2012 to 2017 was 30.3 per 1000 person-years.^[3]^ The incidence of shoulder pain is known to correlate with job-related mechanical exposure such as repetitive motion work or work involving awkward postures and vibration.^[1]^

Shoulder pain can cause sleep problems and reduce sleep quality by making sleep difficult to induce or maintain, and changing body position during sleep may also make shoulder pain worse.^[4]^ Sleep decreases and relaxes shoulder muscle tone; thus, a decrease in the amount and quality of sleep could affect the relaxation and recovery of shoulder muscle.^[5]^ After all, the cycle runs on repeat when sleep problems are caused by shoulder pain and shoulder pain worsens due to lack of quality sleep, and worse shoulder pain can eventually cause shoulder dysfunction.

Psychological stress including job stress can increase muscle tension and activity, which can increase shoulder pain.^[6,7]^ Also, psychological factors such as depression and anxiety may influence shoulder pain intensity, and inversely, shoulder pain may affect psychological factors.^[8]^ Since people with high levels of pain perceive their pain as a threat,^[8]^ high levels of shoulder pain can increase depressive symptoms, stress, and anxiety. In particular, patients with depressive and anxious temperament have been reported to have a high risk of committing suicide;^[9,10]^ thus, active efforts are needed to solve shoulder pain and psychological problems.

In general, women have a higher incidence of shoulder pain than men, and women’s psychological stress in particular is closely related to shoulder pain.^[3]^ Shoulder pain is known to have the highest incidence between the ages of 50 and 59, however, shoulder pain is gradually increasing in young people due to decreased physical activity, lack of exercise, increased computer work and increased use of smartphones.^[11,12]^

In order to reduce shoulder pain and shoulder disability, factors related to shoulder pain have been analyzed^[1,8]^ and various interventions^[11,13]^ have been attempted, however effective methods have not yet been developed. This may be because shoulder pain is caused by a combination of personal and psychological factors as well as the work environment. In other words, a multifaceted analysis of shoulder pain and shoulder disability in consideration of various factors should be necessary to develop an intervention method to reduce shoulder pain and shoulder disability. In women particularly, psychological stress and sleep problems are highly related to shoulder pain,^[3]^ thus identifying the multifaceted relationship among sleep quality, psychological factors such as depression, and anxiety, and shoulder disability with shoulder pain will be effective in developing intervention programs for women.

Mediation analysis can determine the effects of a third variable in the pathway between an independent and a dependent variable, and multiple mediation analysis can consider multiple mediators/confounders simultaneously.^[14]^ Therefore, this study aims to investigate the association between shoulder pain and shoulder disability based on a multiple mediation model. The relative influence and structural causality of shoulder pain, sleep quality, and psychological factors on shoulder disability in women were examined.

Our specific objectives were as follows: first, to describe the levels of depression, anxiety, stress, shoulder pain, shoulder disability, and sleep quality in women; second, to identify the association between depression, anxiety, stress, shoulder pain, shoulder disability, and sleep quality in women; and thirdly, to identify the mediating effects of sleep quality and depression in the relationship between shoulder pain and disability in women.

## 2. Methods

### 2.1. Study design and sample size

**Figure 1.**
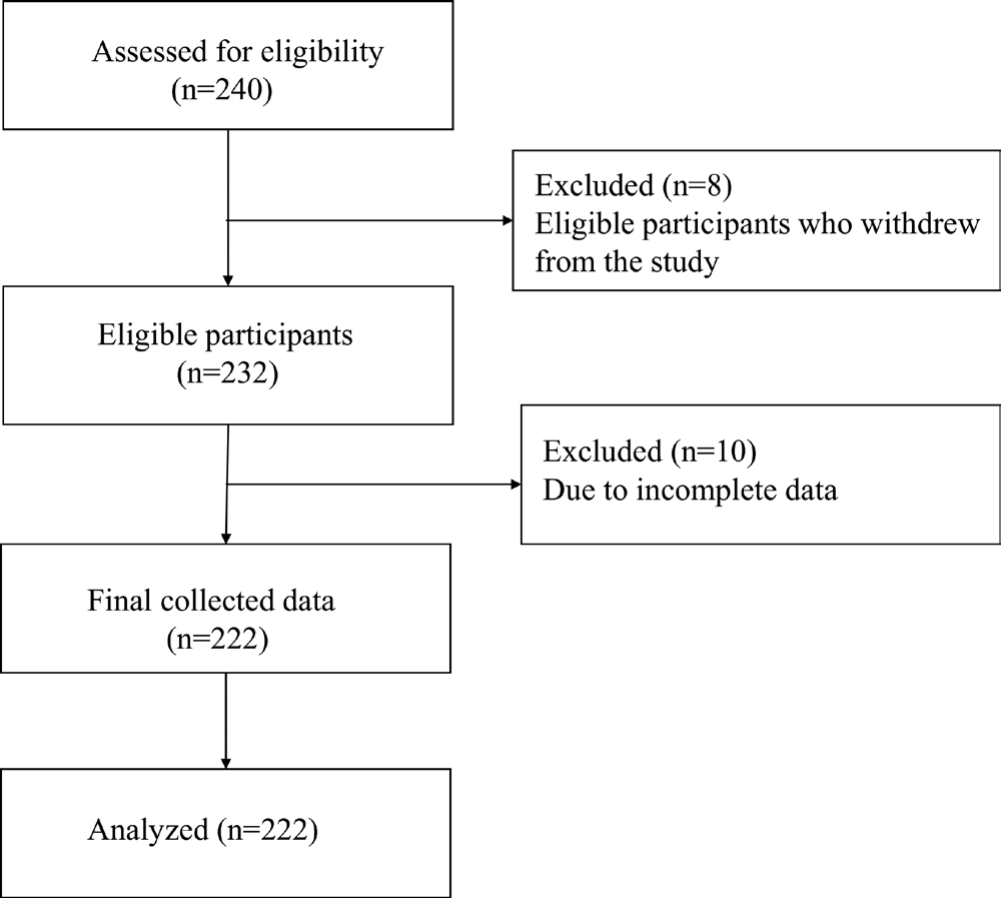
Flow chart of the study.

This study was conducted on female participants ranging from 35 to 64 years of age and residing in Seoul and Gyeonggi-do regions, with an aim to identify the serial multiple mediation effects of sleep quality and psychological factors such as depression, anxiety, and stress and the influence of shoulder pain on shoulder disability. Questionnaires were distributed to a total of 240 subjects who visited 3 community centers in Seoul and Gyeonggi-do regions. Ten individuals who provided incomplete responses and 8 individuals who dropped out of the study were excluded. Finally, the data of 222 participants were analyzed (Fig. 1).

The present investigators made a direct visit to the 3 community centers to provide a face-to-face explanation on the study purpose and protocol to the participants. Thereafter, a signed written consent was obtained from each individual who expressed voluntary agreement to participate. The time taken to complete the survey was approximately 20 minutes and the survey consisted of a self-reporting questionnaire.

The sample size was estimated using the G POWER 3.1 program. On performing power analysis under the conditions of medium effect size = 0.15, power = 0.95, and significance level of α = 0.05, the minimum number of samples was calculated to be 199. Considering the dropout rate, the sample size of n = 222 was thus adequate.

The data was collected from May 4, 2021 to June 23, 2021. The inclusion criteria for the study were as follows: women in the age range of 35 to 64 years and could communicate and complete a self-reported survey. We excluded participants who were unable to communicate either or have intellectual disability, participants who were unable to give informed consent, participants who provided an ambiguous general response, and participants who provided incomplete or partial answer.

### 2.2. Depression anxiety stress scale-21 (DASS-21)

DASS-21 was used to measure the levels of depression, anxiety, and stress.^[15]^ DASS-21 is a tool measuring the frequency of signs or symptoms of depression, anxiety, and stress in the past weeks. The tool is a self-reporting questionnaire on a 4-point scale with a score of 0 (“does not apply to me at all”) to a score of 3 (“applies to me most of the time”). It consists of 21 questions in total with 7 questions in each of the 3 subcategories of depression, anxiety, and stress. The total score ranges from the minimum of 0 to the maximum of 63 based on the 3 subcategories, each with the score ranging from 0 to 21. The increase in the score indicates the increase in the severity of depression, anxiety, or stress. At the time the tool was developed, the Cronbach’s α coefficients for depression, anxiety, and stress were reported as 0.91, 0.84, and 0.90, respectively.^[13]^ In this study, the Cronbach’s α coefficients for depression, anxiety, and stress were uniformly high at 0.90, 0.89, and 0.88, respectively, indicating good internal consistency.

### 2.3. Shoulder pain and disability index (SPADI)

For the assessment of levels of shoulder pain and shoulder disability, the SPADI, developed by Roach et al (1991) and revised by Seo et al^[16]^ for suitability to Korean subjects, was used. The Korean version of SPADI consisted of 5 questions on the pain subscale for the assessment of pain in individuals and 8 questions on the function/disability subscale for the measurement of the level of shoulder dyskinesia. All 13 questions were based on the 10-cm visual analogue scale, with the score of 0 indicating “No pain” or “No distress” and the score of 10 indicating “Severe pain” or “Mandatory assistance.” The total score in each subscale was converted to percentage with 0% indicating a perfectly healthy state and 100% indicating the worst state possible. Hence, the increase in the score indicates the increase in the severity of pain or disability. At the time of tool development, the total Cronbach’s α was 0.95 with the Cronbach’s α for pain = 0.86 and the Cronbach’s α for disability = 0.95.^[17]^ The Cronbach’s α in this study was high at 0.94, indicating high reliability.

### 2.4. Sleep quality

To evaluate the quality of sleep in the participants, the one question was used: “How would you evaluate your overall sleep quality in the past month?” The question was on a 4-point scale; the score of 1 indicated “very good” and the score of 4 indicated “very poor”; no other questions were used.

### 2.5. Ethical considerations

The study plan and process were approved by the Clinical Ethics Committee of the Daejeon University in accordance with the principles of the Helsinki Declaration (1040647-202006-HR-013-02). Eligible participants were approached and invited to participate. Eligible participants signed a written consent before the start of the survey after researchers provided participants with a detailed explanation of the purpose and method of the study directly. The participants were informed that the participation in the study is voluntary, that they will not be at any disadvantage during the participation in the study, and that they can withdraw participation at any time if they wish. The survey was distributed only to participants who agreed to participate, and the collected data were used for study purposes only. The guarantee of anonymity and autonomy of study participants was described. The collected survey was stored in a secure cabinet.

### 2.6. Statistical analyses

The descriptive statistics and Pearson’s *r* correlation were performed with the SPSS statistical version 23.0 (IBM SPSS Statistics for Windows, IBM Corp., Armonk, NY). To verify the serial mediating effect of sleep quality and DASS-21 (depression, anxiety, stress) in the relationship between shoulder pain and shoulder disability, Hayes’s Model 6^[18]^ of the PROCESS macro for SPSS was used for analysis. In serial mediation, mediating factors (sleep quality and DASS-21) are expected to affect the shoulder disability directly and indirectly. The serial mediation model is a useful study model to identify the precedence between 2 mediating variables in the relationship between shoulder pain and shoulder disability. The bootstrapping process of SPSS PROCESS macro was used to estimate the mediating effects in this study. Using random sampling, 10,000 samples were generated, and a 95% bias-corrected confidence interval (BC CI) was employed for analysis of the mediating effects. If the 95% CI excluded zero, the effect was considered significant.

## 3. Results

### 3.1. Participant general characteristics

Table 1 displays the general characteristics of the participants. Of a total of 240 participants responding to the survey, 18 individuals who dropped out or provided insincere responses were excluded, and data from 222 participants were analyzed in this study (response rate was 92.5%). The age range of the participants was 35 to 64 years, while the mean age was 44.27 (6.98) years and regarding ages, most of the participants were shown by individuals aged 35 to 39 years (62.2%). Of the total, the percentage of married individuals was greater at 71.6%, nondrinkers and nonsmokers were 52.7% and 95%, respectively, and individuals not performing regular exercise were 56.8%. For the level of education, the percentage of individuals with an undergraduate degree was the highest at 81.1%. For the level of income, those in the “middle” level showed the highest percentage at 78.8%. The percentage of individuals without a diagnosed chronic disease was 58.1% and that of individuals with 1 chronic disease was 36.9%.

**Table 1:** General characteristics (N = 222).

Variable	Category	*n* (%)	Mean (SD)
Age (yrs)	35–39	138 (62.2)	44.27 (6.98)
(35–64)	40–49	62 (27.9)	
	≥50	22 (9.9)	
Marital status	Single	49 (22.1)	
	Married	159 (71.6)	
	Divorced or widowed	14 (6.3)	
Alcohol	Yes	105 (47.3)	
	No	117 (52.7)	
Smoking	Yes	11 (5.0)	
	No	211 (95.0)	
Exercise	Yes	96 (43.2)	
	No	126 (56.8)	
Education	≤High school	42 (18.9)	
	≥College	180 (81.1)	
Economic status	High	10 (4.5)	
	Middle	175 (78.8)	
	Low	37 (16.7)	
No. of chronic diseases	0	129 (58.1)	
	1	82 (36.9)	
	≥2	11 (5.0)	

SD = standard deviation.

### 3.2. Scores for DASS-21 depression, anxiety, stress, shoulder pain, shoulder disability and sleep quality

As shown in Table 2, the average score for sleep quality was 2.31 (0.642), the average score obtained using DASS-21 depression was 4.04 (3.97), DASS-21 anxiety was 3.83 (3.85) and DASS-21 stress was 5.45 (4.11). The average score for shoulder pain subscale was 20.07 (13.09) and shoulder disability subscale was 19.77 (18.00).

**Table 2:** Levels of sleep quality, DASS-21, and SPADI (N = 222).

Variables	Range	Mean (SD)
Sleep quality	1–4	2.31 (0.642)
DASS-21		
DASS-21 depression	0–21	4.04 (3.97)
DASS-21 anxiety	0–21	3.83 (3.85)
DASS-21 stress	0–21	5.45 (4.11)
SPADI pain subscale	0–50	20.07 (13.09)
SPADI disability subscale	0–80	19.77 (18.00)

DASS-21 = depression anxiety stress scale-21, SD = standard deviation, SPADI = shoulder pain and disability index.

### 3.3. Correlations between depression, anxiety, stress, shoulder pain, shoulder disability, and sleep quality

Pearson’s correlations between variables are presented in Table 3. It was found that the DASS-21 depression had a positive correlation with DASS-21 anxiety (*R* = 0.861, *P* < .001), DASS-21 stress (*R* = 0.837, *P* < .001), shoulder pain subscale (*R* = 0.230, *P* < .001), shoulder disability subscale (*R* = 0.329, *P* < .001), and sleep quality (*R* = 0.189, *P* = .005). DASS-21 anxiety was positively and significantly associated with DASS-21 stress (*R* = 0.852, *P* < .001), shoulder pain subscale (*R* = 0.314, *P* < .001), shoulder disability subscale (*R* = 0.417, *P* < .001), and sleep quality (*R* = 0.223, *P* = .001). And DASS-21 stress was positively and significantly associated with shoulder pain subscale (*R* = 0.299, *P* < .001), shoulder disability subscale (*R* = 0.335, *P* < .001), and sleep quality (*R* = 0.311, *P* < .001). And shoulder pain subscale was positively and significantly associated with shoulder disability subscale (*R* = 0.764, *P* < .001) and sleep quality (*R* = 0.286, *P* < .001). And shoulder disability subscale was positively and significantly associated with sleep quality (*R* = 0.259, *P* < .001).

**Table 3:** Correlation among variables (N = 222).

Variables	r (*P*)					
	DASS-21			SPADI		Sleep quality
	1	2	3	4	5	6
1. Depression	―					
2. Anxiety	0.861 (<.001)	―				
3. Stress	0.837 (<.001)	0.852 (<.001)	―			
4. Pain subscale	0.230 (.001)	0.314 (<.001)	0.299 (<.001)	―		
5. Disability subscale	0.329 (<.001)	0.417 (<.001)	0.335 (<.001)	0.764 (<.001)	―	
6. Sleep quality	0.189 (.005)	0.223 (.001)	0.311 (<.001)	0.286 (<.001)	0.259 (<.001)	―

DASS-21 = depression anxiety stress scale-21; SPADI = shoulder pain and disability index.

### 3.4. The mediation effects of sleep quality and depression in the relationship between shoulder pain and disability

Table 4 presents the result of the bootstrap test (bootstrap sample: 10,000) through the Hayes’s SPSS macro PROCESS to analyze the serial multiple mediation effects of sleep quality and depression in the relationship between shoulder pain and disability. The result indicated that depression had a mediating effect in the relationship between shoulder pain and disability. The total effect of shoulder pain on shoulder disability was significant (B = 1.0541, SE = 0.0611, t = 17.2467, *P* < .001). The direct effect of shoulder pain on shoulder disability was also significant (B = 1.0028, SE = 0.0636, t = 15.7665, *P* < .001). With the addition of sleep quality and depression as mediating variables, the effect of shoulder pain on shoulder disability was significant although decreased. Notably, the total indirect effect of shoulder pain on shoulder disability through the mediation of sleep quality and depression was shown to be significant (B = 0.0513, SE = 0.0333, 95% BC CI [0.0082, 0.1216]). The result thus suggested that the indirect as well as the direct effects of the 2 mediating variables (sleep quality and depression) was significant enough to imply partial mediation effects of sleep quality and depression in the relationship between shoulder pain and disability.

**Table 4:** Total, direct, and indirect effects in a multiple mediator model (N = 222).

Model	Effect	SE	t	*P*	95% BC CI
**Mediated variable (M2): DASS-21 Depression**					
Total effect of shoulder pain on shoulder disability	1.0541	0.0611	17.2467	<.001	[0.9936, 1.1746]
Direct effect of shoulder pain on shoulder disability	1.0028	0.0636	15.7665	<.001	[0.8773, 1.1282]
Total indirect effect	0.0513	0.0333			[0.0082, 0.1216]
Indirect effect via					
Sleep quality	0.0001	0.0278			[-0.0520, 0.0584]
Sleep quality and depression	0.0414	0.0219			[0.0075, 0.0917]
Depression	0.0098	0.0066			[-0.0004, 0.0258]
**Mediated variable (M2): DASS-21 Anxiety**					
Direct effect of shoulder pain on shoulder disability	0.9713	0.0640	15.1780	<.001	[0.8451, 1.0974]
Total indirect effect	0.0828	0.0377			[0.0159, 0.1576]
Indirect effect via					
Sleep quality	-0.0029	0.0278			[-0.0554, 0.0534]
Sleep quality and anxiety	0.0729	0.0299			[0.0242, 0.1394]
Anxiety	0.0128	0.0084			[0.0007, 0.0331]
**Mediated variable (M2): DASS-21 Stress**					
Direct effect of shoulder pain on shoulder disability	1.0079	0.0654	15.4005	.001	[0.8789, 1.1369]
Total indirect effect	0.0462	0.0311			[-0.0089, 0.1133]
Indirect effect via					
Sleep quality	-0.0019	0.0286			[-0.0568, 0.0589]
Sleep quality and stress	0.0363	0.0210			[0.0001, 0.0828]
Stress	0.0118	0.0078			[0.000, 0.0301]

BC CI = bias-corrected confidence interval, DASS-21 = DEPRESSION ANXIETY STRESS Scale-21, SE = standard error.

**Figure 2.**
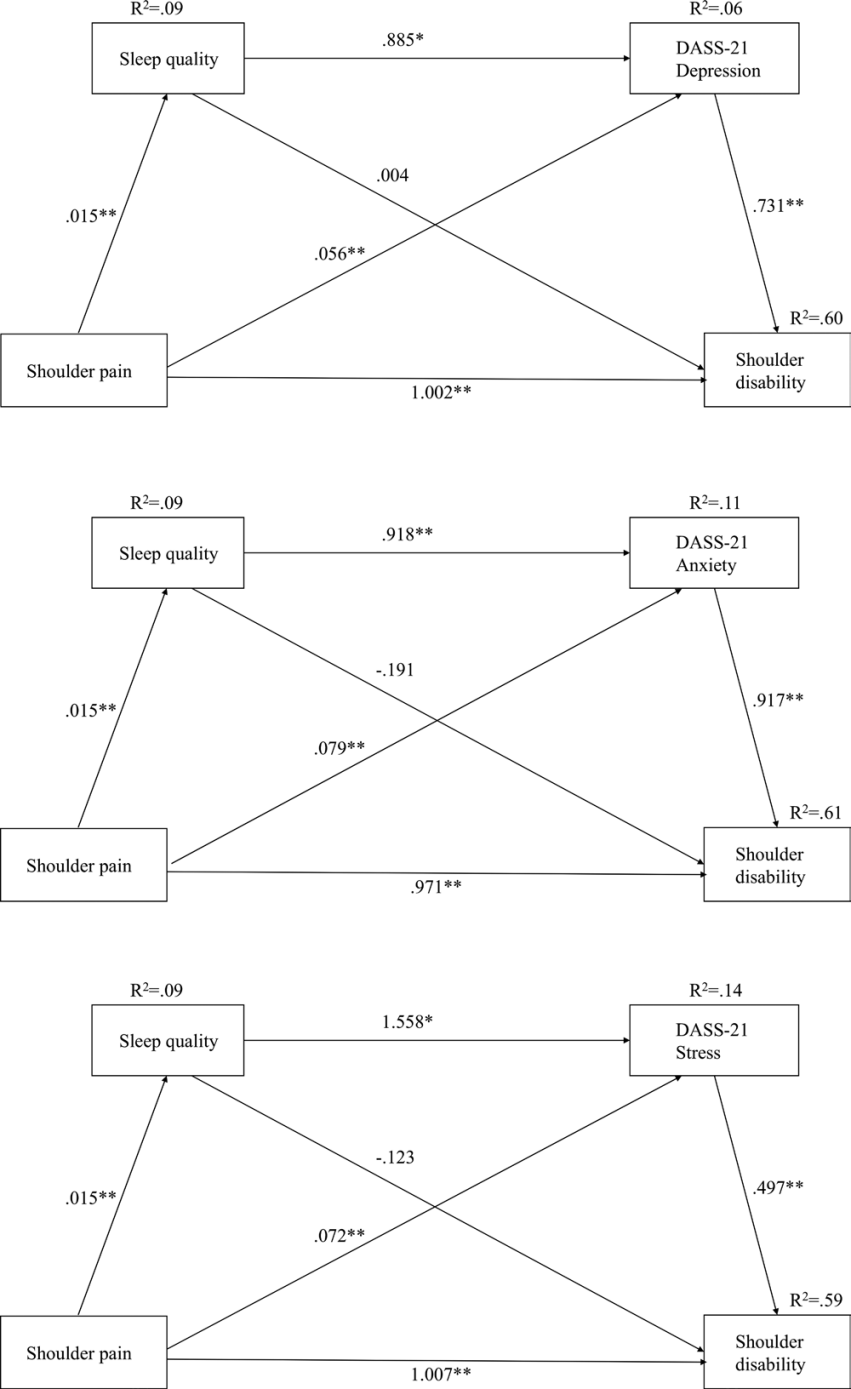
Tests of serial mediation model showed the indirect effect of sleep quality, and the sequential indirect effects of the sleep quality and psychological disorders (DASS-21 depression, anxiety, and stress), in the association between shoulder pain and shoulder disability.

In addition, the bootstrapped indirect effect of shoulder pain on shoulder disability through sleep quality and depression as mediating variables was significant (B = 0.0513, SE = 0.0333, 95% BC CI [0.0082, 0.1216]). However, the direct effect of shoulder pain on shoulder disability through the mediation of sleep quality or depression was not significant (CIs included zero). Figure 2 shows the standardized path coefficients of the proposed serial multiple mediation model, indicating the direct path coefficient between shoulder pain and shoulder disability. Based on the result, the explanatory power of the overall model of shoulder disability variables was 60%. This indicated that an increase in shoulder pain led to an increase in the severity of shoulder disability and that the pain caused a drop in sleep quality, while a high level of depression increased the severity of shoulder disability. Hence, the control of shoulder pain is predicted to lead to enhanced sleep quality and reduced depression leading towards an improvement in shoulder disability.

### 3.5. The mediation effects of sleep quality and anxiety in the relationship between shoulder pain and disability

Analyzing the mediating effects of sleep quality and anxiety in the relationship between shoulder pain and disability showed that the direct effect of shoulder pain on shoulder disability was significant (B = 0.9713, SE = 0.0640, 95% BC CI [0.8451, 1.0974]) and the total indirect effect of shoulder pain on shoulder disability through the mediation of sleep quality and anxiety was significant (B = 0.0828, SE = 0.0377, 95% BC CI [0.0159, 0.1576]). The result suggested that the 2 mediating variables (sleep quality and anxiety) had a significant indirect as well as direct effects to imply partial mediation effects of sleep quality and anxiety.

In addition, the bootstrapped indirect effect of shoulder pain on shoulder disability through sleep quality and anxiety as mediating variables was significant (B = 0.0828, SE = 0.0377, 95% BC CI [0.0159, 0.1576]). In the relationship between shoulder pain and disability, the indirect effect through the mediation of sleep quality and anxiety was significant (B = 0.0729, SE = 0.0299, 95% BC CI [0.0242, 0.1394]) and the indirect effect through the mediation of anxiety was significant (B = 0.0128, SE = 0.0084, 95% BC CI [0.0007, 0.0331]). Based on the result, the explanatory power of the overall model of shoulder disability variables was 60%. This indicated that an increase in shoulder pain caused a drop-off in sleep quality due to pain, while a high level of anxiety increased the severity of shoulder disability.

### 3.6. The mediation effects of sleep quality and stress in the relationship between shoulder pain and disability

Analyzing the mediation effects of sleep quality and stress in the relationship between shoulder pain and disability showed that the direct effect of shoulder pain on shoulder disability was significant (B = 1.0079, SE = 0.0654, 95% BC CI [0.8789, 1.1369]) and the total indirect effect of shoulder pain on shoulder disability through the mediation of sleep quality and stress was significant (B = 0.0462, SE = 0.0309, 95% BC CI [-0.0093, 0.1126]). The results suggest that the 2 mediating variables (sleep quality and stress) had significant indirect as well as direct effects adequate to imply partial mediation effects of sleep quality and stress.

In addition, the total indirect effect of shoulder pain on shoulder disability through sleep quality and stress as mediating variables was not significant (CIs included zero). However, in the relationship between shoulder pain and disability, the indirect effect through the mediation of sleep quality and stress was significant (B = 0.0363, SE = 0.0206, 95% BC CI [0.0022, 0.0804]). The indirect effect through the mediation of stress was significant (B = 0.0118, SE = 0.0077, 95% BC CI [0.0005, 0.0302]). But the indirect effect through the mediation of sleep quality was not significant (CIs included zero). Based on the results, the explanatory power of the overall model of shoulder disability variables was 59%. This indicated that an increase in shoulder pain caused a dip in sleep quality due to pain, while a high level of stress increased the severity of shoulder disability.

## 4. Discussion

Recent studies have considered the effect of shoulder pain not only on shoulder disability^[19]^ and sleep quality;^[4]^ but also on psychological factors such as depression, anxiety, and stress.^[8]^ This study used a multiple mediation model to show that shoulder pain and shoulder disability have a multifaceted relationship in women.

In this study, shoulder pain directly affected shoulder disability, and the more severe shoulder pain was, the more serious shoulder disability was in women. This result is consistent with findings of previous studies^[19,20]^ that shoulder pain causes shoulder dysfunction by reducing shoulder joint movement. As severity of shoulder pain is associated with shoulder disability,^[19,20]^ interventions to reduce shoulder pain will be effective in decreasing shoulder disability. Various interventions such as therapeutic exercise, cognitive behavioral approach, and acupuncture therapy have been developed to reduce shoulder pain.^[21–23]^ If the interventions can be customized for suitability to each specific subject, these will be effective in reducing shoulder pain and shoulder disability.

Sleep quality has a mediating effect on the relationship between shoulder pain and shoulder disability in women, and can be explained by the results from Canivet et al, Tekeoglu et al, and Park et al^[4,5,24]^ Shoulder pain can cause arousal from sleep, interfering with the initiation and maintenance of sleep, thereby shoulder pain can decrease the quality of sleep.^[24]^ Sleep may be a time when the shoulder muscles are relaxed and recoverable, however, if the quality of sleep is poor, the shoulder muscles cannot relax and recover, which may cause shoulder disability during the day.^[5,24]^ Therefore, it can be predicted that shoulder disability can be effectively reduced if a method of improving sleep quality is applied alongside interventions that reduce shoulder pain.

It is particularly interesting that psychological factors such as anxiety, depression, and stress were also partial mediators in the relationship between shoulder pain and shoulder disability in this study. This result is supported by work from previous studies that reported shoulder pain as more frequent in people with psychological problems, especially among women, and that shoulder disability is associated with psychological problems.^[3,8,25,26]^ Persistence of high-intensity shoulder pain may facilitate the disuse of the affected shoulder and lead to abnormal defensive behavior to avoid the shoulder pain, which can affect daily life activities including sleeping or driving a vehicle. Therefore, people with shoulder pain can perceive of pain as a threat or fear of life and experience symptoms of depression, anxiety, and stress. Inversely, psychological problems may also cause more shoulder disability.^[8,27]^ It will be necessary to consider psychological factors when developing interventions to reduce shoulder pain and shoulder disability for women. When women with shoulder pain have a psychological problem such as depression, anxiety, or stress, an intervention that can solve the psychological problem should be considered, and it is necessary to monitor whether new psychological problems occur.

Lastly, the result of this study, that severity of shoulder pain can affect shoulder disability through sleep quality and psychological factors such as depression, anxiety, and stress in women, is supported by the result of Karatel et al^[28]^ Therefore, it may be beneficial to add to the treatment of women with shoulder pain a psychiatric evaluation or an emphasis on improving sleep quality.^[28,29]^ In particular, since people with affective temperaments have a high risk of suicide,^[10]^ if the psychological problems are exacerbated by chronic shoulder pain, chronic shoulder pain can lead to suicidal behavior. Therefore, this study emphasizes the importance of reducing shoulder pain before the onset of psychological problems and quality of sleep. In addition, this study provides a theoretical basis for developing interventions to reduce shoulder pain in consideration of sleep quality and psychological problems.

There are some limitations to this study. First, as this is a cross-sectional study, the direction of the causal relationship among variables was identified using a statistical method that can be investigated at the level of a cross-sectional study. Thus, longitudinal studies are needed to confirm the results. It will be necessary to identify changes in shoulder pain, sleep quality, psychological problems, and shoulder disability over time and to determine the causal relationships among them. Second, additional variables that may affect shoulder disability have not been considered. In the future, it will be necessary to investigate the effects on shoulder disability using other variables. Third, this study used self-reported data from women; therefore, there is a possibility of selection bias in which the data does not correctly reflect respondents’ attitudes. Finally, the generalizability of this study was limited because the data were collected in 3 community centers located in South Korea using convenience sampling.

Despite these limitations, this study provides new insight into the multifaceted relationship between shoulder pain, sleep quality, psychological factors, and shoulder disability, which provided the evidence for considering sleep quality and psychological factors when developing interventions to reduce shoulder pain and shoulder disability for women.

Shoulder pain management of women is a critical determinant factors of shoulder disability. Thus, in nursing practice, the effective assessment and management of shoulder pain should be prioritized in women with shoulder pain. Moreover, determinants of pain (e.g., spoor sleep, depression, anxiety, and stress) may negatively affect shoulder disability in women.

## 5. Conclusion

Recently, research has been conducted that shoulder pain interacts with various factors such as shoulder dysfunction, sleep quality, and psychological problems.^[28,29]^ This study aimed to identify the association between severity of shoulder pain and shoulder disability using a multiple mediation model in women. The direct effect of severity of shoulder pain on shoulder disability was statistically significant. The effect of serial multiple mediation on predicting shoulder disability from severity of shoulder pain, sleep quality, and psychological factors such as depression, anxiety, and stress was significant, accounting for 60% of the variance in shoulder disability. In women, shoulder pain can reduce the quality of sleep and cause psychological disorders, and the poor sleep quality and psychological disorders can further affect shoulder dysfunction. Therefore, it is necessary to carefully observe whether poor sleep quality and psychological disorders occur in women with shoulder pain. And if a woman with shoulder pain experiences poor sleep quality and psychological disorders, it may be necessary to develop a customized program that can mitigate shoulder pain, poor sleep quality, and psychological disorders all together.

## Author contributions

**Conceptualization:** Younghui Hwang.

**Data curation:** Younghui Hwang.

**Writing – original draft:** Jihyun Oh.

**Writing – review & editing:** Jihyun Oh.
